# Quantifying the Increase in Radiation Exposure Associated with SPECT/CT Compared to SPECT Alone for Routine Nuclear Medicine Examinations

**DOI:** 10.1155/2011/897202

**Published:** 2011-07-04

**Authors:** Ann M. Larkin, Yafell Serulle, Steven Wagner, Marilyn E. Noz, Kent Friedman

**Affiliations:** Department of Radiology, School of Medicine, New York University, 550 First Avenue, New York, NY 10016, USA

## Abstract

*Purpose*. We quantify the additional radiation exposure in terms of effective dose incurred by patients in the CT portion of SPECT/CT examinations. *Methods*. The effective dose from a variety of common nuclear medicine procedures is calculated and summarized. The extra exposure from the CT portion of the examination is summarized by examination and body part. Two hundred forty-eight scans from 221 patients are included in this study. The effective dose from the CT examination is also compared to average background radiation. *Results*. We found that the extra effective dose is not sufficient to cause deterministic effects. However, the stochastic effects may be significant, especially in patients undergoing numerous follow-up studies. The cumulative effect might increase the radiation exposure compared to patient management with SPECT alone. *Conclusions*. While the relative increase in radiation exposure associated with SPECT/CT is generally considered acceptable when compared with the benefits to the patient, physicians should make every effort to minimize this effect by using proper technical procedures and educating patients about the exposure they will receive.

## 1. Introduction

Since the introduction of helical computed tomography (CT) in the early 1990s, the rapid development of the technology and capabilities of these scanners has accelerated the implementation of many new clinical applications. One such advantage is the hybrid imaging SPECT/CT system, which is continuing to gain popularity in nuclear medicine and cardiology facilities. The clinical advantages of SPECT/CT compared to SPECT alone have been documented by several authors [1–4]. Combined SPECT/CT systems allow the sequential acquisition of anatomic and molecular data while minimizing changes in patient position, improving attenuation correction, and providing inherently coregistered anatomic images. 

Although the technology clearly has its benefits, new developments in hybrid imaging technology, including combining SPECT with multislice CT systems, result in higher exposure levels for patients, a greater occupational hazard to staff from scattered radiation and the requirement for increased shielding in a nuclear medicine department. In general, effective dose (E) for CT examinations can be higher than most other diagnostic imaging modalities [5]. There is also a considerable choice of CT user-selectable exposure factors resulting in a significant variation in CT dose to the patient. In addition, in a nuclear medicine facility devoid of CT technologists, there could potentially be limited knowledge of CT techniques, clinical applications, and associated dose consequences. In recent years the radiation dose incurred by CT examinations has been a subject of much concern; several authors have highlighted the need for dose optimization in CT, specifically in paediatric patients [6–8]. According to 2006 data, approximately 62 million CT examinations were performed in hospitals and out-patient facilities in the United States [9], and the number of CTs continues to grow by 10–15% per year [10]. Thus, CT will continue to contribute to a significant portion of the total collective dose delivered to the public from medical procedures involving ionizing radiation. This study aims to characterize the additional radiation exposure associated with the CT portion of SPECT/CT for a selection of common nuclear medicine procedures, to assist clinicians in meeting the guide lines presented by the International Commission on Radiation Protection [6] with respect to minimizing the effective dose necessary to achieve beneficial results for the patient. 

## 2. Materials and Method

Patient data from a dual-headed SPECT unit with an integrated 6-slice CT scanner (Symbia T6 E-Cam, Siemens Medical Systems, Erlangen, Germany) is presented in this study. This CT portion of this system has variable tube current (20–345 mA), slice thickness of 0.6–10 mm, and rotational speed of 0.6–1.5 secs. The tube voltage for the clinical portion of the acquisition was 130 kVp. 

Data from 248 scans, comprising 221 patients is presented in this study. The contribution of total effective dose imparted by the nuclear tracers for each patient was calculated by multiplying the average administered activity for all patients by the “effective dose per unit administered activity” conversion factors listed in the International Commission on Radiological Protection (ICRP) Publication 53 [11] and 80 [12]. 

 The effective dose from the CT portion of the examination is calculated from the product of the dose length product (DLP) and a body-region-specific conversion factor, *k* (mSv mGy^−1^ cm^−1^), which takes into account the varying biological sensitivities of different organs as given in Table 3 of AAPM report 96 [8]. DLP is a patient-specific value determined by the scan length and the acquisition parameters; it represents the total amount of radiation delivered in the acquisition. 

The CT scan was acquired immediately following completion of the SPECT study with the patient in the same position to minimize motion errors. Acquisition of patient examinations on the Symbia T6 were performed using a tube current modulation system, Care Dose 4D (Siemens). CARE Dose 4D adjusts the tube current over the patient's *z*-axis (one dimension) and in the *x*-axis and *y*-axis (three dimensions) based on the topogram. Real-time four-dimensional modulation is available during scanning and is controlled by using feedback from the previous rotation to set the tube current according to the attenuation measured at each tube angle [13]. The intention of this modulation is to select the appropriate tube current to achieve a predetermined level of image noise, independent of patient size and anatomic configuration. 

In accordance with quality control (QC) regulations, standard body and head CT phantoms are used to measure the weighted computed tomography dose index (CTDIw) on an annual basis. This parameter is then used to calculate the CTDIvol, which is a measure of the average dose within the scan volume to a standardized phantom. The DLP is then calculated from the product of the CTDIvol and the scan length. The phantoms employed in the QC are a 32 cm diameter CT body phantom and a 16 cm CT head and neck phantom; both phantoms are 15 cm thick and are composed of solid acrylic (Capintec, USA).

## 3. Results and Discussion

The QC phantom-measured DLP for head and body phantoms were in tolerance and compared well to the DLP displayed on the console postacquisition (body: −4.3%, head: −1.6%).

Table 1 displays the whole body effective dose as a result of the radiopharmaceutical administered for a range of routine nuclear medicine examinations analyzed in this study.  Table 2 displays the average effective dose for the CT portion of the exam. Table 3 displays the average effective dose for the CT portion of the exam, based on the anatomical region being scanned.

The purpose of the CT scan in a SPECT/CT system is to provide improved attenuation correction and to obtain precise anatomic localization of radiopharmaceutical distributions. It is generally accepted that these objectives can be achieved using CT scans with a much lower exposure level than that of standard diagnostic CTs. The Symbia T6 is a diagnostic-quality CT and as such has the capability of acquiring scans in the exposure range of a standard diagnostic CT. The values obtained on this system are similar to that of a diagnostic CT. For SPECT/CTs with diagnostic CT capabilities such as the Symbia T6, there is the risk that unnecessary high levels of radiation will be used. 

There are two possible explanations as to why the SPECT/CT dose is similar to that of a diagnostic CT. The first could be that lower exposure levels are being employed, but the field of view of the SPECT/CT (~40 cm) is larger than the standard diagnostic abdomen examination (~25 cm), resulting in a larger volume of tissue being scanned. As the SPECT and CT are acquired in succession, before the SPECT is reconstructed and analyzed, it may not be possible to optimize the CT scan region unless information pertaining to the location of suspicious patient anatomy is available prior to the scan. The lack of this information may result in the acquired CT scan length to be, by default, similar in length to that of the SPECT scan (40 cm FOV). This could result in organs being exposed unnecessarily. 

Alternatively, even though dose modulation is employed on this system, the target noise level could be set at a level that results in a high effective mAs and “diagnostic” image quality. Sufficient localization information may be obtained with a lower radiation dose to the patient. It may be possible to increase the target noise level, thereby reducing the effective mAs for SPECT/CT examinations. 

Table 4 displays the effective dose for the SPECT portion of the examination and the CT effective dose. In general only one CT per examination is preformed except in the case of the ProstaScint examination, where the patient undergoes an abdomen and a pelvis CT. However, depending upon variable clinical scenarios, some patients may undergo more than one SPECT/CT scan, either to image more than one part of the body (e.g., chest and abdomen/pelvis) or to followup at later time points in order to improve accuracy of scan interpretation.

Patient dose may be compared with background radiation exposures from natural sources which in the United States averages 3 mSv per year [8]. In the results presented in Table 5 the corresponding background radiation in years is given for the average effective dose for each SPECT/CT procedure.

The radiation dose levels measured here are not sufficient to result in deterministic effects. The principal risks to patients at these dose levels are the stochastic process of carcinogenesis and genetic effects [15]. Hall and Giaccia estimate that the risk of fatal cancers increases by 4% for every Sv of total effective dose received by a patient. For the Siemens system, the increased risk of fatal cancer by the inclusion of an abdomen CT is 0.03%. In the context of one study this may not represent significant carcinogenic risk, but, in patients undergoing numerous follow-up studies, the cumulative effect could result in a significant increase in radiation exposure compared to patients previously managed with SPECT alone. However, it is worth noting that before the availability of SPECT/CT patients frequently have been sent for additional radiological examinations (X-ray or CT) subsequent to the SPECT examination; thus, additional radiation exposure would have occurred in these cases as well. If a diagnostic level CT is acquired during the SPECT/CT examination, it may negate the need for a separate diagnostic CT, if required.

The risk figures presented here only give an estimate of the risk involved for the CT portion of the SPECT/CT examination. The effective dose metric is useful in comparing dose from different diagnostic procedures and for comparing radiation risks associated with different technologies. However, in order to determine an accurate risk to any individual the absorbed dose to irradiated tissues is the more appropriate quantity.

## 4. Conclusion

Despite the advantages associated with SPECT/CT, questions remain regarding its broad applicability to all traditional SPECT applications. Physicians with hybrid cameras are encouraged to carefully consider the risks and benefits associated with adding CT to their SPECT studies prior to imaging a patient. Understanding the risks and benefits of SPECT/CT versus SPECT alone is important not only for deciding on what type of scan to perform but also in order to properly educate patients regarding the exposure they will receive during a test. In general, the relative increase in radiation exposure associated with SPECT/CT compared to SPECT alone is considered acceptable given the potential benefits associated with this technique. However, every effort should be made to adhere to the “As Low as Reasonably Achievable (ALARA)” principle and ensure that the patient is not subjected to unnecessarily high levels of radiation.

## Figures and Tables

**Table 1:** Whole body effective dose as a result of the radiopharmaceutical administered for a range of routine nuclear medicine examinations.

Study type	Average radiopharmaceutical activity (MBq)	Effective dose per unit activity (mSv/MBq) [11]	Average radiopharmaceutical effective dose (mSv)
^67^Gallium	370	0.1	37.0
^99m^Tc-MDP	1110	0.0057	6.3
^99m^Tc-MIBI parathyroid	925	0.009	8.3
^111^In-Octreotide	222	0.054	12.0
^111^In-WBC	18.5	0.36	6.7
^ Tc99^m Ceretec	740	0.0093	6.9
^131^I Uptake	74	0.52	38.5
^131^I Posttherapy Scan	5550	0.52	2886.0
^111^In Chloride (ProstaScint)	222	0.15	32.4

**Table 2:** Average effective dose for the CT portion of the routine nuclear medicine examinations.

Study type	No. of patients	Average effective dose (mSv)	Range effective dose (mSv)
^67^Gallium	10	8.2 ± 5.0	0.4–18.8
^99m^Tc-MDP	17	3.8 ± 3.9	0.2–12.4
^99m^Tc-MIBI parathyroid	78	5.4 ± 1.7	0.7–8.5
^111^In-Octreotide	4	7.0 ± 3.3	3.7–11.1
^111^In-WBC	4	6.4 ± 4.1	1.4–10.9
^ Tc99^m Ceretec	2	15.1 ± 0.4	14.8–15.4
^131^I Uptake	35	5.6 ± 2.8	0.5–12.3
^131^I Posttherapy Scan	32	6.8 ± 3.1	1.1–12.8
^111^In Chloride (ProstaScint)	39	9.5 ± 4.9	3.6–23.3

**Table 3:** The average effective dose for the CT portion of the exam, based on the anatomical region being scanned.

CT scan region	No. of scans	CT ED (mSv)	Average effective dose from a diagnostic CT [14]
Chest	40	7.4 ± 2.7	7
Abdomen	41	8.6 ± 4.6	8
Pelvis	67	6.1 ± 2.0	6
Head	10	0.7 ± 0.5	2
Neck	81	5.2 ± 1.8	3
Head and neck	9	1.2 ± 0.7	—

**Table 4:** The percentage increase in the effective dose by examination by including the CT.

Study type	Average radiopharmaceutical effective dose (mSv)	CT effective dose (mSv)	% increase in effective dose by the inclusion of the CT
^67^Gallium	37.0	8.2	22%
^99m^Tc-MDP	6.3	3.8	60%
^99m^Tc-MIBI parathyroid	8.3	5.4	65%
^111^In-Octreotide	12.0	7	58%
^111^In-WBC	6.7	6.4	96%
^ Tc99^m Ceretec	6.9	15.1	219%
^131^I Uptake	38.5	5.6	15%
^131^I Posttherapy Scan	2886.0	6.8	<1%
^111^In Chloride (ProstaScint)	32.4	9.5	29%

**Table 5:** Effective dose and equivalent background radiation for the total SPECT/CT effective dose for each examination.

Study type	Total effective dose (mSv)	Equivalent background radiation (years)
^67^Gallium	45.2	15.1
^99m^Tc-MDP	10.1	3.4
^99m^Tc-MIBI parathyroid	13.7	4.6
^111^In-Octreotide	19.0	6.3
^111^In-WBC	13.1	4.4
^ Tc99^m Ceretec	22.0	7.3
^131^I Uptake	44.1	14.7
^131^I Posttherapy Scan	2892.8	964.3
^111^In Chloride (ProstaScint)	41.9	14.0
